# A Call for a Reform of the Influenza Immunization Program in Mexico: Epidemiologic and Economic Evidence for Decision Making

**DOI:** 10.3390/vaccines9030286

**Published:** 2021-03-19

**Authors:** Roberto Tapia-Conyer, Miguel Betancourt-Cravioto, Alejandra Montoya, Jorge Abelardo Falcón-Lezama, Myrna María Alfaro-Cortes, Rodrigo Saucedo-Martínez

**Affiliations:** 1 Vaccinology Section, Sociedad Mexicana de Salud Pública, Mexico City 11590, Mexico; tapiaconyer@yahoo.com.mx (R.T.-C.); betancom70@gmail.com (M.B.-C.); airain.montoya@gmail.com (A.M.); myrna.alfaro.fcs@gmail.com (M.M.A.-C.); rosaucedo@gmail.com (R.S.-M.); 2 School of Medicine, Universidad Nacional Autónoma de México, Mexico City 04510, Mexico

**Keywords:** influenza, vaccination, immunization, cost-effectiveness, burden of disease, Mexico, middle-income countries

## Abstract

Limited information is available to determine the effectiveness of Mexico’s national influenza vaccination guidelines and inform policy updates. We aim to propose reforms to current influenza vaccination policies based on our analysis of cost-effectiveness studies. This cross-sectional epidemiological study used influenza case, death, discharge and hospitalization data from several influenza seasons and applied a one-year decision-analytic model to assess cost-effectiveness. The primary health outcome was influenza cases avoided; secondary health outcomes were influenza-related events associated with case reduction. By increasing vaccination coverage to 75% in the population aged 12–49 years with risk factors (diabetes, high blood pressure, morbid obesity, chronic renal failure, asthma, pregnancy), and expanding universal vaccination coverage to school-aged children (5–11 years) and adults aged 50–59 years, 7142–671,461 influenza cases; 1–15 deaths; 7615–262,812 healthcare visits; 2886–154,143 emergency room admissions and 2891–97,637 hospitalizations could be prevented (ranges correspond to separate age and risk factor groups), with a net annual savings of 3.90 to 111.99 million USD. Such changes to the current vaccination policy could potentially result in significant economic and health benefits. These data could be used to inform the revision of a vaccination policy in Mexico with substantial social value.

## 1. Introduction

Every year, influenza affects millions of people of all ages around the world, which results in an economic burden of approximately 87,100 million (95% confidence interval (CI): 47,200–149,500) US dollars (USD) due to direct and indirect costs, which include healthcare visits, hospitalization days and productivity loss due to the disease [1]. In Latin America, the annual incidence of influenza-like illness (ILI) ranges from 4.7% to 15.4% [2]. Worldwide, influenza cases are estimated to result in three to five million cases of severe disease and 290,000–650,000 deaths [3]. Influenza vaccination is one of the most effective interventions to prevent the transmission of the disease and to reduce the risk of death and severity of influenza-related complications such as pneumonia, bronchitis, sinus or ear infections, and exacerbation of chronic conditions in all age groups [4,5]. However, some population groups may be more susceptible to transmission and to developing more severe disease, particularly those suffering from comorbidities [6,7].

In 2004, the influenza vaccine became part of the Mexican Universal Vaccination Program. As of 2010, Mexico’s national vaccination schedule recommends yearly influenza immunization in several target groups: children aged 6 to 59 months, adults aged ≥ 60 years, pregnant women, at-risk individuals aged 5 to 59 years and health professionals [8]. These target groups are eligible to receive the vaccine free of charge at any public health facility during the influenza immunization season (October to April); people not included in these groups can only access influenza vaccines in the private sector. Vaccine coverage rates within the aforementioned groups included in the vaccination program vary between 9.9% (95% CI, 8.2–11.9) and 33.9% (95% CI, 32.3–35.4) [9].

The current official immunization guidelines are due for review as they were last revised in 2010 after the influenza A H1N1 pandemic [8]. Therefore, a sound body of evidence is needed to understand the epidemiology and impact of the influenza vaccine on the burden of disease and to guide necessary adjustments and enhancements to these guidelines, which will result in economic and health benefits.

Mexico’s current influenza vaccination guidelines exclude age groups whose coverage has been demonstrated as cost-effective in other countries [10,11,12,13]. Such age groups include school-aged children 5 to 11 years old and adults 50 to 59 years old with no risk factors. From the societal perspective, inclusion of these groups would be highly beneficial since the former has one of the highest incidence rates and risk of transmission to the general population [13,14,15,16,17,18,19], and the latter group exhibits the highest mortality rates attributable to influenza within an economically active population, which has a significant impact on loss of productivity [10,11]. Among the age groups currently included in the immunization program, those aged 5 to 59 years old with risk factors show a low vaccination coverage, despite having the highest susceptibility to influenza-related complications and death [5,15,20].

The objective of this paper is to provide guidance to the national influenza vaccination policy in Mexico. Our proposal for policy reform is based on our analysis of the health and economic benefits of expanding the current national influenza vaccination program using an integrated strategy. We analyzed an expansion strategy for three populations: expanding universal vaccination coverage to all school-aged children (5 to 11 years), increasing vaccination coverage to 75% in people aged 12 to 49 years with risk factors (diabetes mellitus, uncontrolled hypertension, morbid obesity (body mass index ≥40 kg/m^2^), chronic renal failure (CRF), asthma, pregnancy), and expanding universal vaccination coverage to all adults aged 50 to 59 years. Our analysis is based on the findings of two previous cost-effectiveness studies conducted by the authors of the present manuscript; one of which has been published and the other is in press [21,22].

## 2. Materials and Methods

In this paper, we generate a series of public policy recommendations on the basis of epidemiological and economic analysis of expanding vaccination coverage for influenza in Mexico, whose methodologies can be found in previous publications [21,22]. We conducted a two-phase study. First, the burden of disease (infections, hospitalizations, deaths, lethality, potential lost life years (PLLY), and presence of risk factors) was characterized based on the confirmed cases of influenza in Mexico and their healthcare costs during the 2009–2010 to 2017–2018 influenza seasons. Lethality rate was defined as the proportion of deaths among people having a confirmed case of influenza, whereas PLLY was defined as the estimate of the average years a person would have lived if this person had not died prematurely, which was based on the age group of a deceased person. Next, a cost-effectiveness analysis was conducted for a typical one-year season for which the health and economic benefits were estimated upon implementing the following three strategies: (1) expanding universal vaccination to all school-aged children (5 to 11 years); (2) increased vaccination coverage of the population aged 12 to 49 years with risk factors to 75% and (3) expanding universal vaccination to all adults aged 50 to 59 years.

The primary information sources were the Influenza Epidemiological Surveillance System (SISVEFLU, as per its acronym in Spanish) [23], the Epidemiological and Statistical Registry of Mortality (SEED, as per its acronym in Spanish) [24], and the Automated Hospital Discharge System (SAEH, as per its acronym in Spanish) [25]. Additionally, population estimates from the National Population Council (CONAPO, as per its acronym in Spanish) [26] were considered for age group breakdowns and projections, and data projected by the US Centers for Disease Control and Prevention (CDC) were used for influenza incidence rates through indirect standardization [27]. For the purposes of the study, the case definition of influenza was taken from the current Mexican guidelines for epidemiological surveillance and treatment of influenza [28]. Hospital discharges and influenza-associated deaths were coded according to the International Statistical Classification of Diseases and Related Health Problems, 10th Revision (Supplementary Text S1) [29].

### 2.1. Epidemiological Analysis

For the epidemiological characterization of confirmed influenza cases in the SISVEFLU database, the morbidity and mortality patterns were described for each season of the study period for both A and B-type circulating viruses in order to outline the trend and seasonality by epidemiological week. Hospital discharge information from the SAEH was used to estimate the number of hospitalizations and average length of stay due to influenza-associated causes.

### 2.2. Economic Analysis

**Figure 1:**
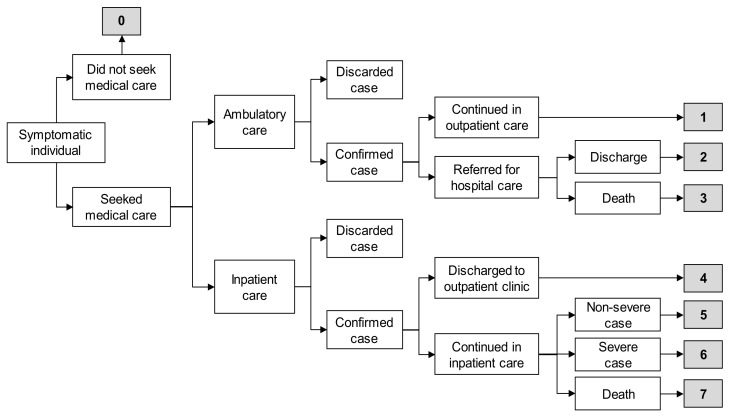
Scenarios for economic evaluation [21]. Based on data from the Influenza Epidemiological Surveillance System (SISVEFLU, as per its acronym in Spanish), eight health outcome scenarios were considered for estimating the costs of influenza cases. We took into consideration if patients did not request medical care (scenario 0), sought ambulatory care (scenarios 1 through 3), or sought hospital care (scenarios 4 through 7). Scenario 0: Symptomatic individual did not seek medical care, self-medicated with over-the-counter drugs, and had a complete recovery. Scenario 1: Symptomatic individual visited an outpatient clinic, had a positive polymerase chain reaction (PCR) result for influenza, was managed only in ambulatory care, and had a complete recovery. Scenario 2: Symptomatic individual visited an outpatient clinic, had a positive PCR result for influenza and was referred for hospital care due to severity, had a complete recovery, and was discharged. Scenario 3: Symptomatic individual visited an outpatient clinic, had a positive PCR result for influenza and was referred for hospital care due to severity, and died. Scenario 4: Symptomatic individual visited a hospital emergency room (ER), had a positive PCR result for influenza, was discharged to an outpatient clinic for follow-up, and had a complete recovery. Scenario 5: Symptomatic individual visited a hospital ER, had a positive PCR result for influenza, was admitted to hospital for follow-up with non-severe clinical status, and had a complete recovery. Scenario 6: Symptomatic individual visited a hospital ER, had a positive PCR result for influenza, was admitted for follow-up with severe clinical status, and had a complete recovery. Scenario 7: Symptomatic individual visited a hospital ER, had a positive PCR result for influenza, was admitted to hospital for follow-up, and died.

The characterization of the economic analysis was thoroughly described previously [21]. To assess the cost effectiveness of expanding the influenza vaccination schedule, a decision-tree analytical model was developed [30]. Eight scenarios were created based on SISVEFLU clinical data regarding the type of care influenza patients received (outpatient or inpatient care), diagnostic test results and treatment outcome (recovery, complication, or death). The probability of occurrence was estimated for each scenario (Figure 1).

Influenza cases were estimated based on the incidence published by the United States CDC [28], which was applied to the Mexican population structure [26] for each group of interest: (1) school-aged population (5 to 11 years old) [21], (2) population aged 12 to 49 years with risk factors, and considering the six most frequent conditions and/or the influenza-related conditions with the highest impact on the health of the Mexican population: diabetes mellitus, uncontrolled hypertension, CRF, morbid obesity, pregnancy and asthma [31,32,33], and (3) population aged 50 to 59 years without risk factors [22]. Each at-risk population group was studied separately without considering the possibility of comorbidities. Therefore, the groups cannot be pooled together to estimate the overall prevalence of risk factors in the population aged 12 to 49 years and should be analyzed independently as there is no way to assess patients with combined comorbidities.

To determine the size of the population aged 12 to 49 years with risk factors, we obtained the prevalence rates of the six most frequent conditions and/or the influenza-related conditions with the highest impact on the health of the Mexican population from national surveys: diabetes mellitus, uncontrolled hypertension, CRF, grade III or morbid obesity (body mass index ≥ 40 kg/m^2^), pregnancy and asthma (calculated based on the prevalence rates by age group as reported in national surveys [24,25,29] and Table 1 and Table 2).

Estimated cases were allocated into the different scenarios, applying the probability of occurrence for each scenario to distribute the cases and to estimate the direct (laboratory diagnosis, medical consultations, drugs, days of hospitalization) and indirect costs (days of medical disability leave, years of life lost) [21] according to the data recorded in SISVEFLU regarding the treatment provided and the guidelines set forth in the Clinical Practice Guideline [34] (Table 3).

Regarding direct and indirect costs, we considered laboratory diagnosis, medical consultations, drugs and days of hospitalization to be direct costs; indirect costs were based on days of medical disability leave and years of life lost. Terms are defined as follows.

For laboratory diagnosis, confirmation of influenza is based on real-time polymerase chain reaction results. In primary health care monitoring units, sample collection for confirmation is only required for 10% of cases, whereas 100% of cases are subject to mandatory sample collection for confirmation in secondary and tertiary health care monitoring units [31]. Throat swab culture is recommended for cases with suspected bacterial coinfection.

Regarding medical consultations, cases detected and managed in outpatient clinics (scenario 1) require two medical consultations; the first for clinical diagnosis and prescription of treatment, and the second to confirm complete recovery. Patients admitted to the hospital via an outpatient clinic (scenario 2) require one ambulatory consultation and two specialty consultations, the first to begin treatment at the hospital and the second at discharge. For scenario 3 (admission to hospital via an outpatient clinic that results in death), we considered one initial ambulatory consultation where clinical diagnosis and referral occurs and three specialty consultations at the hospital. An emergency consultation was mandatory for all hospital-managed patients admitted through the emergency room (ER) (scenarios 4–7). In these scenarios, patients had one, two, three and four medical consultations in scenarios 4, 5, 6, and 7 respectively, assuming a proportional increase in the number of medical consultations with the disease severity.

Regarding drugs, for individuals not requesting medical care (scenario 0) and only requiring over-the-counter drugs, we assumed the use of amantadine for influenza treatment and paracetamol for acute pain management. For all confirmed cases, either ambulatory or inpatient care, we assumed the prescription of oseltamivir for influenza treatment and paracetamol for acute pain management. The use of ceftriaxone was assumed for antibiotic treatment in patients with a bacterial coinfection (scenarios 2, 3, 5, 6, and 7).

Regarding days of hospitalization, for patients admitted via an outpatient clinic who were referred for hospitalization and later discharged (scenario 2), we assumed a one-day hospital stay, mostly for monitoring of symptoms and clinical evolution. For patients admitted via an outpatient clinic who were referred for hospitalization that resulted in death (scenario 3), we considered the average hospital stay (8.3 days) for patients with influenza [32]. Very importantly, we assumed that a hospital stay is provided independently of the admitting area or laboratory confirmation of the case, and that those unit costs are already accounted for. Patients admitted to the hospital for observation via the ER who were discharged for follow-up at an outpatient clinic (scenario 4) were considered to have had a two-day hospital stay to monitor their progress until discharge. Patients with non-severe cases admitted via the ER for medical care (scenario 5) were considered to have the average 8.3-day hospital stay. For patients with severe cases admitted to hospital via the ER (scenario 6), we assumed a 50% longer hospital stay (12.45 days) than patients with nonsevere cases. Finally, for patients admitted via the ER whose outcome was death (scenario 7), we assumed the average hospital stay of 8.3 days.

Days of medical leave were estimated based on the guidelines of the Mexican Institute of Social Security. For patients diagnosed in outpatient clinics without hospital admission (scenario 1), a three-day medical leave was assumed. For patients diagnosed in outpatient clinics with a hospital referral (scenario 2), a seven-day medical leave was assumed after a one-day hospital stay, for a total of eight days of leave. For patients admitted via the ER and later discharged for follow-up at an outpatient clinic (scenario 4), a three-day medical leave was assumed after a two-day hospital stay, for a total of five days of leave. For those admitted via the ER and hospitalized as non-severe cases (scenario 5), a seven-day medical leave was assumed after an eight-day hospital stay, for a total of 15 days of absence. For patients admitted via the ER and hospitalized as severe cases (scenario 6), a 14-day medical leave was assumed after a 12-day hospital stay, for a total of 26 days of absence [33].

For the calculation of years of life lost, the age of each influenza-confirmed death registered in SISVEFLU was considered individually. The lower limit was the age of one year for all subjects and the upper limit was the ages of 73 and 78 years for males and females, respectively, following Mexico’s current life expectancy. These ages were later weighed according to the population distribution by sex and discounted using a 5% discount rate.

The determination of unit costs per item, including the vaccination cost and the weighted average cost of affiliation of the population per season, was based on official information sources described in a previous publication [21] (Table 4); cost estimates are reported in US dollars.

To estimate direct medical costs, we considered public unit costs of each of the institutions that comprise the Mexican Health System, and then weighed them by the proportion of the population affiliated with each institution for the influenza seasons from 2009–2010 to 2018–2019. Absenteeism due to influenza was estimated using the average daily wage of an individual (obtained from the 2018 National Survey of Household Income and Expenditure) [26]. Finally, costs associated with premature deaths were projected depending on the age of an individual at the time of their death, and costs were discounted using the World Health Organization’s recommended 5% discount rate.

Costs were originally obtained in Mexican pesos (MXN) and later converted to 2018 constant prices using the National Consumer Price Index published by Mexico’s National Bureau of Statics and Geography. Data are presented in USD using the average exchange rate published in the Official Federal Gazette between January 2019 and August 2019 (USD 1 = MXN 19.2155).

Mexico currently uses the trivalent inactivated influenza vaccine in the national immunization program. The price per dose of influenza vaccine was obtained from Mexico’s Ministry of Health for 2018 (MXN 57.68, USD 3.00), whereas the cost of administration (MXN 4.54, USD 0.24) and the cost of transportation and storage (MXN 0.55, USD 0.03) were obtained from Gutierrez and Bertozzi’s study and converted to 2019 prices [28].

Each of the three expansion strategies was evaluated considering the reduction in the number of influenza cases (including associated deaths) from the perspective of the external payer and society as the primary objective. The reduction in healthcare visits, diagnostic tests, treatments, hospitalizations, sick leave and PLLY derived from such reductions were defined as secondary health outcomes in the study, considering a 5% discount rate according to the World Health Organization guidelines for performing economic analysis [35]. The term of the analysis was a typical one-year influenza season, which was used to reflect the seasonality of the disease. The efficacy of the vaccine was set at 50%, which is the average effectiveness of the influenza vaccine in the northern hemisphere of the Americas as published elsewhere for the influenza seasons from 2009–2010 to 2017–2018 [21,22], while the actual coverage of the influenza vaccine was obtained from national surveys (Table 5 and Table 6). Analyses were done using Microsoft Excel (2013) software.

## 3. Results

### 3.1. Influenza Burden of Disease

**Figure 2:**
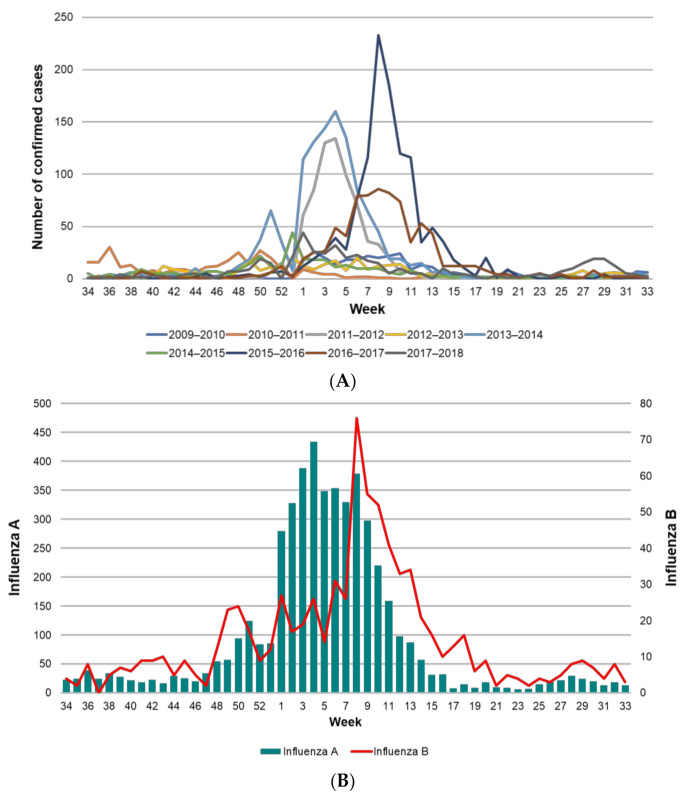
Confirmed influenza cases in Mexico from 2009 to 2018. (**A**) Confirmed cases of influenza per epidemiological week by season (2009–2018); (**B**) cases of influenza type A and B by epidemiological week (2009–2018). Different scales were used for influenza A and B.

Using the surveillance criteria for influenza, SISVEFLU recorded a total of 390,862 suspected cases (Influenza-Like Illness and Severe Acute Respiratory Infection) during the study period, of which 50,900 (13.03%) were confirmed with a diagnostic test. The trend and seasonality observed during the epidemiological weeks that were included in each study season were as expected, with increased activity between week 46 and week 20 of the following year; cases peaked between weeks three and nine (Figure 2). It is worth emphasizing that excess cases were observed during the 2011–2012, 2013–2014, 2015–2016, and 2017–2018 seasons (Figure 2A). Analysis by type of infecting virus showed a bimodal pattern for influenza B, with two peaks during the year at weeks 49 and 8; most cases occurred between weeks 40 and 10. This suggests a longer duration of infectious activity of influenza B compared with influenza A (Figure 2B).

### 3.2. Hospital Discharges and Length of Stay

**Figure 3:**
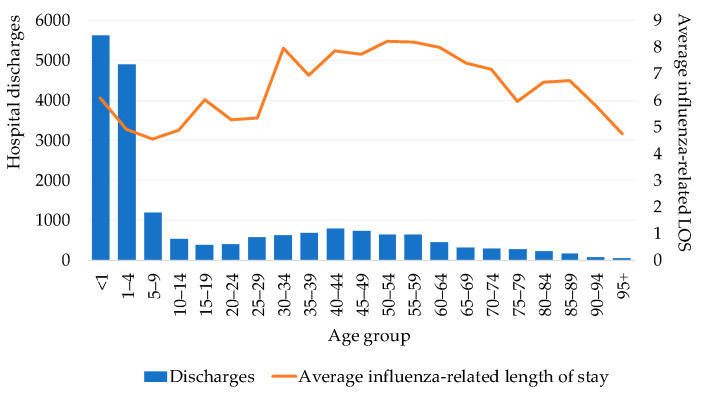
Hospital discharges and average length of stay by age group (2010–2016). LOS = length of stay.

Using SAEH data [25], the number of influenza-related hospital discharges was 8631 during the study period, which represented 56,667 hospital days and an average length of stay of 6.6 hospital bed-days per hospitalized patient (Figure 3). Influenza-related hospital admissions were similar for males and females but were less frequent among young adults compared with children under five years of age and adults over 65 years of age. The longest average length of stay was seen in the 50 to 59 years old group (8.3 days), followed by the 30 to 34 and 60 to 64 years old groups (8.1 and 8.0 days, respectively). The age groups with the shortest average length of stay were five to nine years old (4.5 days) and 10 to 24 years old (4.7 days) (Figure 3).

### 3.3. Deaths and Lethality

**Figure 4:**
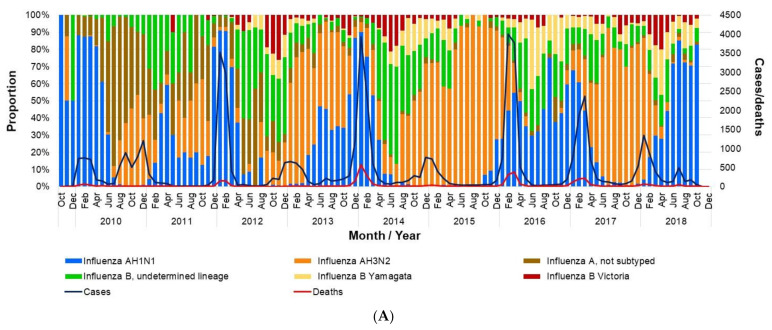

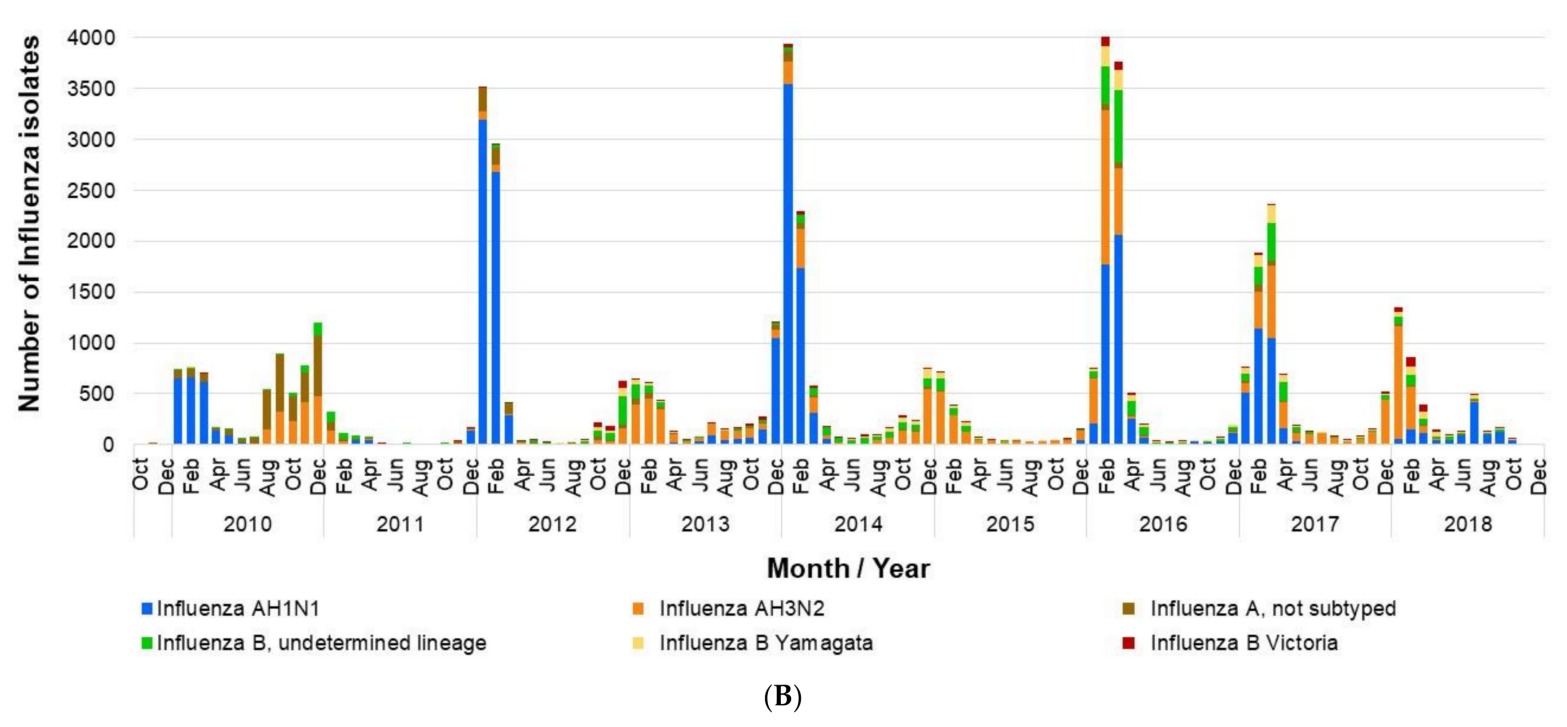
Influenza isolates in Mexico by month (2009–2018). (**A**) Proportion of influenza isolates by type, total cases, and deaths per month; (**B**) number of influenza isolates by type. The data used in this figure were obtained from Mexico’s influenza surveillance system, SISVEFLU.

The number of deaths recorded during the study period was 3717, most of which were associated with influenza A infection. Deaths due to influenza show a strong seasonality with biannual modes, likely influenced by the replacement of strains. They were highest when the AH1N1 subtype was dominant, and lower when AH3N2 was dominant (Figure 4A). Regarding influenza B, the number of isolates (confirmed cases) was low throughout the study period (Figure 4B). Considering the mean life expectancy of the Mexican population, it is estimated that there were 24.5 PLLY for each influenza death, representing a total of 91,124 PLLY during the study seasons.

**Figure 5:**
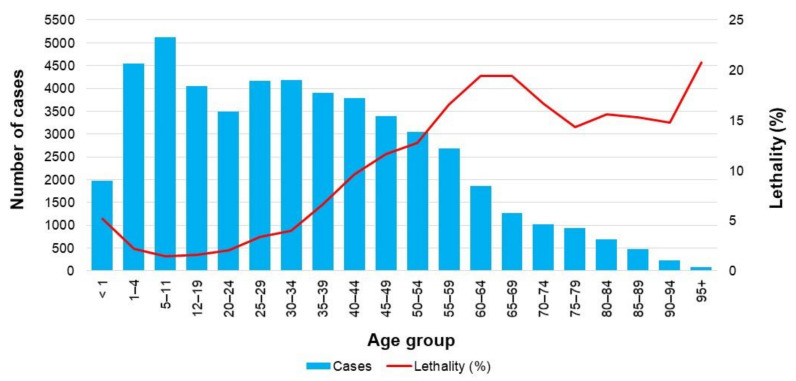
Total number of confirmed cases and lethality rate of influenza (2009–2018). Lethality rate was defined as the proportion of deaths among people having a confirmed case of influenza. The data used in this figure were obtained from Mexico’s influenza surveillance system, SISVEFLU.

The average lethality rate of influenza was 7.3 deaths per 100 confirmed cases. Importantly, the highest number of influenza cases occurred in both the one to four-year-old and five to 11-year-old age groups, and this number tended to decrease with age, while lethality rose significantly after 50 years of age and peaked between 60 and 69 years of age (Figure 5).

### 3.4. Risk Factors in Influenza Cases and Deaths

**Figure 6:**
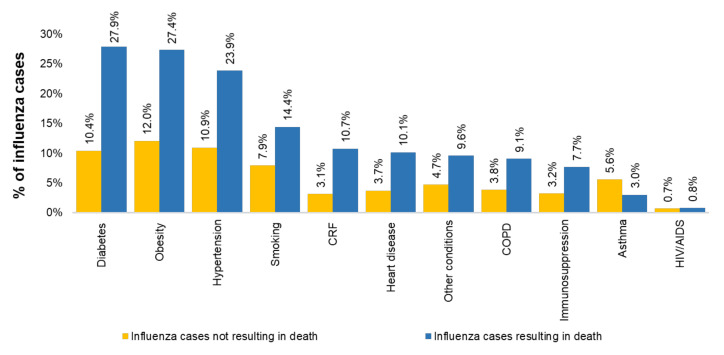
Distribution of observed risk factors. COPD = chronic obstructive pulmonary disease; CRF = chronic renal failure; HIV/AIDS = human immunodeficiency virus-acquired immune deficiency syndrome. The data used in this figure were obtained from Mexico’s influenza surveillance system, SISVEFLU.

Obesity, hypertension and diabetes were the most frequent risk factors in both influenza cases not resulting and resulting in death. The presence of risk factors among the latter was up to three-fold higher than that observed in cases that did not result in death (Figure 6).

### 3.5. Economic Analysis

Assuming a 50% vaccine efficacy, achieving 75% sustained coverage in the population aged 12 to 49 years with risk factors would result in a number of prevented cases of influenza ranging from a minimum of 7142 among individuals with CRF to a maximum of 82,590 among people with diabetes (Table 7).

Regarding the effectiveness of increasing vaccination coverage by age group, for each of the six risk factors analyzed we performed an analysis by age group. First, we estimated the population living with each risk factor based on the estimated prevalence (Table 1). Later, we estimated the total cases of influenza that would occur in a typical season, and later estimated the total cases of influenza for a particular risk factor considering the coverage of the current vaccination program (Table 6). Finally, we estimated the number of influenza cases considering a 75% vaccination coverage, and hence the differential in estimated cases of influenza averted (Table 8, Table 9, Table 10, Table 11, Table 12 and Table 13).

This reduction in influenza cases, in turn, represents a reduction in healthcare visits ranging from 7615 to 92,887; of ER admissions ranging from 2886 to 32,096 and of hospital admissions ranging from 2891 to 32,210. Moreover, the number of deaths prevented would range from 1.1 to 19.4.

This reduction in cases, deaths and associated events would result in an annual net savings between 3.90 and 44.45 million USD, of which between 2.84 and 35.84 million USD would represent direct savings, and between 1.06 and 8.61 million USD would represent indirect savings (Table 14). It should be noted that these estimated savings have accounted for the investment required to expand vaccination coverage, which amounts to 0.5 to 5.5 million USD, depending on the risk group.

Universal expansion of vaccination to both the school-aged (five to 11 years old) population and the age group 50 to 59 years, assuming a 50% vaccination coverage and a 50% vaccine efficacy, would prevent an estimated 870,961 cases of influenza (Table 15). This would result in an annual reduction of 383,610 healthcare visits, 187,167 ER admissions and 130,728 hospital admissions. Additionally, 27 influenza-related deaths would be prevented.

The reduction in cases and deaths would have a positive direct economic impact on both the healthcare system and society. Previous analyses [21,22] showed that expanding universal coverage to these two age groups would result in a net savings of 161.80 million USD, even when considering the 37.95 million USD investment required for the vaccination program. Positive health impacts and economic savings would still be achieved when using a conservative scenario in which vaccination coverage decreases from 50% to 30% and vaccine efficacy decreases from 50% to 19% [21,22].

The cost of influenza-related healthcare was distributed differentially among the groups. The cost of hospital admissions was the most relevant cost in all three expansion strategies (people aged 12 to 49 years with risk factors in Table 14, and children aged five to 11 years and adults aged 50 to 59 years in Table 15). In contrast, indirect cost (sick leave and premature death) represented 8.9% of healthcare cost in the adult population and represented up to a quarter of the healthcare cost for the population aged 5–11 years, mainly due to the high cost associated with premature death in this age group.

## 4. Discussion

### 4.1. Major Findings

Despite the current influenza vaccination policy, a considerable burden of disease persists, which takes a toll on society and amounts to an average of 213.4 million USD per year. This is equivalent to 1.2-fold of the total increase in the public budget allocated to health care services in Mexico in 2020 [36]. Our findings suggest that more than 60% of influenza costs are due to inpatient care, which emphasizes the need for continued reduction of the burden resulting from severe cases and complications of influenza.

We found that a 75% increase in vaccination coverage for any of the risk groups (diabetes, uncontrolled hypertension, morbid obesity, asthma or pregnancy) in the population aged 12 to 49 years would be cost-saving, resulting in savings ranging from 11.7 to 53.6 million USD per year. The benefits of reducing the disease burden would be remarkable. For instance, increasing immunization coverage solely in the population with diabetes would result in 82,590 prevented influenza cases, 92,887 fewer healthcare visits, 32,096 fewer ER admissions, 32,210 fewer hospital admissions and 12.8 fewer deaths. This intervention would be especially relevant to health care services in Mexico since, according to the 2018 National Health Survey, the coverage of influenza vaccination in the risk groups is particularly low, ranging from 9.1% among pregnant women to 35.3% among patients with diabetes mellitus [31], despite the recommendation of 100% vaccination coverage by the Clinical Guidelines as part of healthcare strategies for these at-risk populations [32]. Moreover, epidemiological characterization showed that the prevalence of risk factors was up to three-fold higher among cases that resulted in death. Therefore, this strategy could be among those offering the greatest potential for reducing influenza-related mortality in the Mexican population.

The expansion of universal coverage to the population between 50 and 59 years old without risk factors is widely recommended. It is a cost-effective strategy that would protect a population that is not currently included in the vaccination program but has the highest influenza-related mortality rate in the country (four deaths per 100,000 people) [22]. Moreover, this strategy has proven to be effective in other countries as an alternative to protect the population with unknown chronic underlying conditions [10]. This is relevant because in Mexico, as in most Latin American countries, a high proportion of the population living with chronic conditions is unaware of their health status and would assume to be not covered by the vaccination program. For example, 29% of people with diabetes and 40% with high blood pressure are unaware that they have these conditions [37,38].

Our findings show that the expansion of universal coverage to the school-aged population (five to 11 years) would also be a cost-effective strategy with great potential to reduce the burden of influenza, not only in this particular age but also for the overall population [21]. Currently the school-aged population represents 12.33% of the total population (15.5 million) and has the highest influenza incidence rate (13.9%) and attack rate (20 to 30%) during typical influenza season. Moreover, because influenza spreads faster in indoor environments such as schools, children are considered as “superspreaders” of the disease [18]. Therefore, vaccination of this population has great potential to indirectly protect the remainder of the population by means of herd immunity [13]. This strategy would be one of the most feasible, as the target population can be easily reached through school-based vaccination campaigns.

Even though the ideal scenario would be to achieve universal influenza vaccination coverage for individuals over six months of age, there are operational and financial constraints. Our findings provide evidence that supports the need to at least expand universal coverage to the population aged six months to 11 years and aged 50 to 59 years and ensure sustained vaccination coverage to 75% of the population aged 12 to 49 years with risk factors.

### 4.2. Implications and Recommendations for the Health System

#### 4.2.1. Reinforcing the Coverage of Current Policies

Consistent with reports from other countries [39,40], we presented evidence that to reduce the burden of disease due to influenza in the population aged 12 to 49 years with risk factors, a sustained vaccination coverage of 75% is required. This cost-saving strategy comes with challenges for the health care system, which include ensuring an adequate supply and timely availability of vaccines, as well as the necessary human and financial resources for implementation [40]. In addition to considering the purchase of vaccines, there is need for an integrated approach to purchasing supplies, having properly trained and sufficient healthcare workers, and robust cold chain logistics with adequate inventory management, which may be difficult in middle-income countries. It is also essential to identify and address barriers for vaccination, such as lack of vaccine access and hesitancy to receive the vaccine [39,40]. In other words, public health providers should ensure timely availability of the vaccine, efficient vaccine distribution and the implementation of proactive and effective communication, promotion, training and application strategies for vaccination of the population with risk factors for whom vaccination is recommended. In light of the current COVID-19 pandemic, strategies to promote influenza vaccination, particularly among those with risk factors, are urgently needed, as both the SARS-CoV-2 and influenza viruses cause respiratory illness.

#### 4.2.2. Universal Vaccination to Groups Currently Not Covered

During the past four decades, influenza vaccination efforts in most countries have focused mainly on population groups that have the highest frequency of the most severe and lethal complications [11]. However, programs must have a vision towards universalization. In Mexico, expanding the influenza vaccination program would generate significant health benefits and economic savings from a societal perspective [13,14,15,16,17,18,19]. To this end, the health care system should perform an in-depth feasibility analysis considering technical, funding and programmatic factors [41].

#### 4.2.3. Reinforcing Evidence-Based Decision Making

SISVEFLU currently operates as the main surveillance system through Influenza Health Monitoring Units (USMI, as per its acronym in Spanish). Nonetheless, SISVEFLU only considers a limited number of clinics and hospitals, and this hinders extrapolation of information for the estimation of influenza cases at the national and local levels [23]. The current structure of this platform limits a proper outcome follow-up for each confirmed case. Hence, we propose the adoption of digital health best practices through the deployment of a nominal information system that enables real-time tracking of vaccination coverage, and the timely identification of case occurrence, allowing the implementation of preventive and contention measures to hamper the spread of disease [42,43]. Finally, a nominal information system would allow for systematic and recurrent evaluations of the vaccination program to guide policies and facilitate adjustments whenever necessary.

### 4.3. Limitations

This study was based on administrative and epidemiological surveillance registries; hence, we identified and minimized bias related to inherent quality and data representativeness as much as possible. The economic impact was analyzed using information from SISVEFLU, which, as per design, does not comply with the criteria for presenting a nation as a whole for the estimation of incidence. Therefore, the estimation of cases was based on the incidence rates reported by the Centers for Disease Control and Prevention (CDC), as described elsewhere [21].

When studying the population aged 12 to 49 years with risk factors for influenza complications, we did not include healthcare workers because there is no reliable information about current influenza vaccination coverage in this group.

For economic analysis, costs were estimated using the assumption that there would be no need to create an alternative mechanism for service provision, but rather that the strategies to improve the program would be implemented within the already existing one, an assumption that might underestimate the true cost of interventions. Additionally, official sources of information were used to analyze costs, and we were unable to account for indirect cost variations resulting from socioeconomic or other differences in the population. There is also the issue of comorbidity, which may have led to the overestimation of health and economic savings. We considered six risk factors separately (five comorbid conditions and pregnancy) and calculated the savings as if each risk factor corresponded to a single patient. However, as there are pathological associations, especially between diabetes mellitus, obesity, and hypertension, and in the case of a single patient having two or more concomitant risk factors, the savings would correspond to the cost of a single patient rather than two or more patients. In the present analysis, calculations were made to assess the individual pathologies separately, leading to an overestimation of savings. Finally, we recognize the absence of cost data from vaccine-related adverse events as a limitation of the study.

### 4.4. Relevance of the Study

To our knowledge, this is the first nationwide study conducted in the Latin America region to perform in-depth analyses of the epidemiology of influenza, its healthcare-related costs and the cost-effectiveness of a policy reform. Our results apply specifically to Mexico but are relevant for middle-income countries. Therefore, the present findings could contribute to the evaluation of current public health policies in other countries in the Latin America region and provide evidence and recommendations for strengthening vaccination programs by highlighting the core challenges to enhancing such programs.

Despite the advances in the region, vaccination policies still have important weaknesses; hence there is a need to identify and implement strategies to improve upon these shortcomings [44]. The authors hope that the results of this study will contribute, as a first approach, to applicable middle and long-term strategies in the Latin America region by way of a regionally coordinated health policy aimed towards joint progress and reduction of inequalities of vaccination programs among the countries of the region.

## 5. Conclusions

Our findings show that expanding universal vaccination coverage to all school-aged children (five to 11 years), increasing coverage in the population aged 12 to 49 years with risk factors, and expanding universal vaccination coverage to all adults aged 50 to 59 years could potentially result in significant health and economic benefits. These findings could be used as a major source of evidence for improving the Mexican vaccination policy. For that to be possible, the health care system should perform an in-depth feasibility analysis considering technical, funding and programmatic factors. Expanding vaccine access and making efforts to increase vaccine coverage are expected to benefit all levels of society, including vulnerable populations. Such expansion efforts should aim to provide equal access to vaccination as a tool for disease prevention without limitations of sex, socio-economic status and demographical conditions (e.g., the indigenous population with limited access to health care).

## Figures and Tables

**Table 1:** Sources of information on prevalence of risk factors in the Mexican population.

Disease or Risk Factor	Age Group (Years)	Source of Information for the Estimation of Prevalence
Diabetes	20–59	ENSANUT 2018; self-report of previous medical diagnosis with the following question: 3.1 Has any doctor told you that you are diabetic (or have high blood glucose)?
Uncontrolled hypertension	20–59	ENSANUT 2018; self-report of previous medical diagnosis with the following question: 4.1 Has any doctor told you that you have hypertension? Determination of uncontrolled hypertension based on blood pressure measurement during the survey under these criteria: systolic blood pressure ≥ 140 mmHg and diastolic blood pressure ≥ 90 mmHg.
Morbid obesity	20–59	ENSANUT 2018; Body Mass Index (BMI) estimated from weight and height measurements. $BMI=\frac{Weight\;\left(kg\right)}{height^{2}}$ Morbid obesity is considered as BMI ≥40 kg/m^2^.
Chronic renal failure	20–59	ENSANUT 2018; self-report of previous medical diagnosis with the following question: 6.1 Has any doctor ever told you that you have some kind of kidney disease, like kidney failure?
Asthma	5–59	Global Burden of Disease 2017; prevalence of asthma cases was obtained in 2017.
Pregnancy	15–49	SINAC 2017; total number of live births in 2017 was considered (latest year of available data) as *proxy* for pregnancy during the period of interest.

ENSANUT = Mexican National Health and Nutrition Surveys; SINAC = Mexico Births Information System.

**Table 2:** Prevalence of risk factors in different age group populations.

Age Group (Years)	Diabetes	Uncontrolled Hypertension	Morbid Obesity	Chronic Renal Failure	Asthma	Pregnancy
5–9	ND	ND	ND	ND	6.5	ND
10–14	ND	ND	ND	ND	4.5	ND
15–19	ND	ND	ND	ND	4.5	397,395
20–24	0.4	1.1	3.35	0.3	4.1	604,429
25–29	0.7	0.8	4.90	0.4	2.6	514,830
30–34	2.0	1.8	4.94	0.3	2.2	342,685
35–39	4.4	2.7	6.30	1.1	2.3	163,567
40–44	7.3	5.4	6.10	0.5	2.3	38,175
45–49	10.1	8.0	4.83	0.7	2.2	3419
50–54	16.9	12.0	6.96	0.7	2.2	ND
55–59	19.4	12.8	6.40	1.4	2.3	ND
**Total**	6.7	5.1	5.3	1.0	3.2	2,064,500

Data sources are indicated in Table 1.

**Table 3:** Prescribed treatment per scenario.

Scenario	No Medical Care	Outpatient Only	Hospitalization: Referred from Outpatient Clinic		Hospitalization: Admitted through ER			
	0	1	2	3	4	5	6	7
Health Outcome	Not Demanding Medical Care	Outpatient Only	Hospitalization		Outpatient	Hospitalization		
			Discharge	Death		Non- Severe	Severe	Death
Clinical Diagnosis								
Outpatient consultations ^1^		1	1	1				
ER consultations ^1^					1	1	1	1
PCR ^1^		0.1	0.1	0.1	1	1	1	1
Direct costs								
Outpatient consultations ^1^		1						
Specialist consultations ^1^			2	3	1	2	3	4
Amantadine ^2^	1							
Oseltamivir ^2^		1	1	1	1	1	1	1
Paracetamol ^2^	1	1	1	1	1	1	1	1
Bacteriologic culture ^2^			1	1		1	1	1
Ceftriaxone ^2^			1	1		1	1	1
Hospitalization days			1	8.3	2	8.3	12.45	8.3
Indirect costs								
Medical disability days		3	8		5	15	26	
Years of life lost ^3^				X				X

^1^ Data represent the number of consultations. ^2^ Data represent the number of prescriptions, unit purchased (over-the-counter medications) or culture tests ordered. ^3^ Estimation of the indicator (years of life lost) applies only to the populations in the scenarios indicated by X. Abbreviations: ER = emergency room; PCR = polymerase chain reaction. Data are the authors elaboration of data from Clinical Guidelines, Instituto Mexicano del Seguro Social (IMSS), Automated Hospital Discharge System (SAEH), and Influenza Epidemiological Surveillance System (SISVEFLU).

**Table 4:** Average weighted cost by institution (million USD).

Unit Cost	2009–2010	2010–2011	2011–2012	2012–2013	2013–2014	2014–2015	2015–2016	2016–2017	2017–2018
Outpatient consultations	331.74	323.79	332.10	345.34	356.53	367.07	384.25	416.33	443.64
Specialist consultations	528.72	512.30	524.01	544.56	561.67	578.31	606.14	657.76	701.45
ER consultations	362.78	363.93	375.38	389.29	403.43	414.65	429.28	459.08	484.20
Hospitalization days	3147.49	3040.89	3112.41	3240.17	3340.33	3441.46	3618.09	3939.79	4214.67
Amantadine	32.95	34.07	35.47	36.82	38.30	39.34	40.45	42.90	45.00
Oseltamivir	81.18	83.94	87.40	90.72	94.37	96.94	99.67	105.69	110.87
Paracetamol	2.82	2.86	2.96	3.06	3.18	3.26	3.35	3.56	3.73
Ceftriaxone	8.37	8.61	8.95	9.30	9.66	9.93	10.22	10.86	11.40
PCR	1576.29	1641.95	1712.28	1776.73	1849.83	1899.51	1948.37	2059.96	2155.92
Bacteriologic culture	147.42	152.44	158.71	164.75	171.37	176.03	181.00	191.94	201.34

These data were previously reported [22].

**Table 5:** Sources of information for seasonal influenza immunization coverage.

Disease or Risk Factor	Age Group (Years)	Source of Information for Estimation of Immunization Coverage
Diabetes	20–59	ENSANUT 2018; vaccine application self-report in the 2018–2019 season or vaccine application registered in the immunization card between September 2018 and date of survey
Uncontrolled hypertension	20–59	ENSANUT 2018; vaccine application self-report in the 2018–2019 season or vaccine application registered in the immunization card between September 2018 and date of survey
Morbid obesity	20–59	ENSANUT 2018; vaccine application self-report in the 2018–2019 season or vaccine application registered in the immunization card between September 2018 and date of survey
Chronic renal failure	20–59	ENSANUT 2018; vaccine application self-report in the 2018–2019 season or vaccine application registered in the immunization card between September 2018 and date of survey
Pregnancy	15–49	ENSANUT 2018; vaccine application self-report in the 2018–2019 season or vaccine application registered in the immunization card between September 2018 and date of survey
Asthma	5–59	ENSANUT 2018; vaccine application self-report in the 2018–2019 season or vaccine application registered in the immunization card between September 2018 and date of survey. As the survey does not include information on asthma, national prevalence was considered for this purpose

ENSANUT = Mexican National Health and Nutrition Surveys.

**Table 6:** Seasonal influenza immunization coverage in the 12 to 49 years old population with risk factors.

Age Group (Years)	Diabetes	Uncontrolled Hypertension	Morbid Obesity	Chronic Renal Failure	Asthma	Pregnancy
5–9	ND	ND	ND	ND	26.83	ND
10–14	ND	ND	ND	ND	26.83	ND
15–19	ND	ND	ND	ND	26.83	14.6
20–24	34.8	22.2	19.4	19.3	26.83	14.6
25–29	35.7	26.2	47.6	35.6	28.66	10.6
30–34	35.2	33.8	30.8	34.7	27.00	9.3
35–39	33.6	35.2	23.8	26.7	28.35	7.2
40–44	33.5	29.2	38.2	35.2	25.45	9.4
45–49	36.0	40.3	23.7	39.0	27.40	6.6
50–54	33.3	27.2	25.1	28.6	27.25	ND
55–59	38.6	33.0	35.9	49.5	28.44	ND
Total	35.3	32.1	30.7	34.9	27.4	9.1

ND = No data available for these groups. The source for these data is indicated in Table 5.

**Table 7:** Differentials in prevented influenza cases, deaths and associated events resulting from a 75% increase in vaccination coverage among the population aged 12 to 49 years with risk factors (assuming 50% vaccine efficacy).

Risk Factor	Diabetes	Uncontrolled Hypertension	Morbid Obesity	Chronic renal Failure	Asthma	Pregnancy
Current estimated vaccination coverage (%) ^1^	35.3	32.1	30.7	34.9	27.4	9.1
Prevented influenza cases	82,590	65,146	65,362	7142	70,839	50,781
Prevented deaths	12.8	10.4	19.4	5.1	1.7	1.1
Associated events						
Healthcare visits	92,887	73,787	75,201	7615	79,636	54,521
ER admissions	32,096	22,696	19,474	2886	22,539	20,759
Hospitalization	32,210	22,791	19,549	2891	22,571	20,955

^1^ Details of seasonal influenza immunization coverage according to risk factors are provided in Table 6. ER = emergency room.

**Table 8:** Effectiveness of increasing influenza vaccination coverage to 75% compared to current vaccination coverage: results for the population with diabetes by age group.

Age Group (Years)	Total Population 2018–2019	Estimated Prevalence	Population with Risk Factor	Incidence of Influenza (per 100,000)	Estimated Cases of Influenza	Estimated Cases of Influenza with Current Vaccination Coverage	Estimated Cases of Influenza with 75% Vaccination Coverage	Differential of Estimated Cases of Influenza Averted
20–24	10,803,051	0.4	43,212	7050.5	3047	2509	1904	605
25–29	10,174,627	0.7	71,222	7050.5	5022	4135	3138	997
30–34	9,367,670	2	187,353	7050.5	13,209	10,878	8256	2622
35–39	8,848,819	4.4	389,348	7050.5	27,451	22,606	17,157	5449
40–44	8,391,604	7.3	612,587	7050.5	43,191	35,567	26,994	8573
45–49	7,678,041	10.1	775,482	7050.5	54,675	45,025	34,172	10,853
50–54	6,617,806	16.9	1,118,409	12,382.1	138,483	114,041	86,552	27,489
55–59	5,453,295	19.4	1,057,939	12,382.1	130,995	107,875	81,872	26,003
**Total**	67,334,913		4,255,554		416,073	342,636	260,046	82,590

**Table 9:** Effectiveness of increasing influenza vaccination coverage to 75% compared to current vaccination coverage: results for the population with uncontrolled hypertension by age group.

Age Group (Years)	Total Population 2018–2019	Estimated Prevalence	Population with Risk Factor	Incidence of Influenza (per 100,000)	Estimated Cases of Influenza	Estimated Cases of Influenza with Current Vaccination Coverage	Estimated Cases of Influenza with 75% Vaccination Coverage	Differential of Estimated Cases of Influenza Averted
20–24	10,803,051	1.1	121,913	7050.5	8595	7216	5372	1844
25–29	10,174,627	0.8	84,695	7050.5	5971	5013	3732	1281
30–34	9,367,670	1.8	170,517	7050.5	12,022	10,093	7514	2579
35–39	8,848,819	2.7	240,861	7050.5	16,982	14,256	10,614	3643
40–44	8,391,604	5.4	450,734	7050.5	31,779	26,679	19,862	6817
45–49	7,678,041	8.0	613,430	7050.5	43,250	36,308	27,031	9277
50–54	6,617,806	12.0	796,414	12,382.1	98,613	82,786	61,633	21,152
55–59	5,453,295	12.8	698,582	12,382.1	86,499	72,616	54,062	18,554
**Total**	67,334,913		3,177,147		303,713	254,967	189,820	65,146

**Table 10:** Effectiveness of increasing influenza vaccination coverage to 75% compared to current vaccination coverage: results for the population with morbid obesity by age group.

Age Group (Years)	Total Population 2018–2019	Estimated Prevalence	Population with Risk Factor	Incidence of Influenza (per 100,000)	Estimated Cases of Influenza	Estimated Cases of Influenza with Current Vaccination Coverage	Estimated cases of Influenza with 75% Vaccination Coverage	Differential of Estimated Cases of Influenza Averted
20–24	10,803,051	3.3	361,875	7050.5	25,514	21,598	15,946	5651
25–29	10,174,627	4.9	498,108	7050.5	35,119	29,728	21,949	7779
30–34	9,367,670	4.9	462,805	7050.5	32,630	27,621	20,394	7228
35–39	8,848,819	6.3	557,588	7050.5	39,313	33,278	24,571	8708
40–44	8,391,604	6.1	511,642	7050.5	36,073	30,536	22,546	7990
45–49	7,678,041	4.8	370,726	7050.5	26,138	22,126	16,336	5790
50–54	6,617,806	7.0	460,768	12,382.1	57,053	48,295	35,658	12,637
55–59	5,453,295	6.4	349,276	12,382.1	43,248	36,609	27,030	9579
Total	67,334,912	-	3,572,788	-	295,088	249,792	184,430	65,362

**Table 11:** Effectiveness of increasing influenza vaccination coverage to 75% compared to current vaccination coverage: results for the population with renal disease by age group.

Age Group (Years)	Total Population 2018–2019	Estimated Prevalence	Population with Risk Factor	Incidence of Influenza (per 100,000)	Estimated Cases of Influenza	Estimated Cases of Influenza with Current Vaccination Coverage	Estimated Cases of Influenza with 75% Vaccination Coverage	Differential of Estimated Cases of Influenza Averted
20–24	10,803,051	0.3	27,008	7050.5	1904	1572	1190	382
25–29	10,174,627	0.4	40,699	7050.5	2869	2369	1793	575
30–34	9,367,670	0.3	30,913	7050.5	2180	1799	1362	437
35–39	8,848,819	1.1	93,797	7050.5	6613	5459	4133	1326
40–44	8,391,604	0.5	41,119	7050.5	2899	2393	1812	581
45–49	7,678,041	0.7	54,514	7050.5	3844	3173	2402	771
50–54	6,617,806	0.7	48,972	12,382.1	6064	5006	3790	1216
55–59	5,453,295	1.4	74,710	12,382.1	9251	7636	5782	1855
Total	67,334,913		411,732		35,624	29,407	22,264	7143

**Table 12:** Effectiveness of increasing influenza vaccination coverage to 75% compared to current vaccination coverage: results for the population with asthma by age group.

Age Group (Years)	Total Population 2018–2019	Estimated Prevalence	Population with Risk Factor	Incidence of Influenza (per 100,000)	Estimated Cases of Influenza	Estimated Cases of Influenza with Current Vaccination Coverage	Estimated Cases of Influenza with 75% Vaccination Coverage	Differential of Estimated Cases of Influenza Averted
5–9	11,045,962	6.5	718,259	9602.7	68,972	59,523	43,108	16,415
10–14	11,131,856	4.5	500,934	9602.7	48,103	41,513	30,065	11,449
15–19	11,048,379	4.5	497,177	8581.8	42,667	36,822	26,667	10,155
20–24	10,803,051	4.1	437,842	7050.5	30,870	26,641	19,294	7347
25–29	10,174,627	2.6	266,005	7050.5	18,755	16,185	11,722	4464
30–34	9,367,670	2.2	207,886	7050.5	14,657	12,649	9161	3488
35–39	8,848,819	2.3	201,870	7050.5	14,233	12,283	8896	3387
40–44	8,391,604	2.3	190,837	7050.5	13,455	11,612	8409	3202
45–49	7,678,041	2.2	168,602	7050.5	11,887	10,259	7430	2829
50–54	6,617,806	2.2	147,531	12,382.1	18,267	15,765	11,417	4348
55–59	5,453,295	2.3	127,417	12,382.1	15,777	13,616	9861	3755
Total	100,561,110		3,464,359		297,644	256,867	186,027	70,839

**Table 13:** Effectiveness of increasing influenza vaccination coverage to 75% compared to current vaccination coverage: results for pregnant women by age group.

Age Group (Years)	Total Population 2018–2019	Estimated Prevalence	Population with Risk Factor	Incidence of Influenza (per 100,000)	Estimated Cases of Influenza	Estimated Cases of Influenza with Current Vaccination Coverage	Estimated cases of Influenza with 75% Vaccination Coverage	Differential of Estimated Cases of Influenza Averted
15–19	11,048,379	–	361,192	8581.8	30,997	29,587	19,373	10,213
20–24	10,803,051	–	611,532	7050.5	43,116	41,154	26,948	14,207
25–29	10,174,627	–	542,570	7050.5	38,254	36,513	23,909	12,605
30–34	9,367,670	–	363,084	7050.5	25,599	24,435	16,000	8435
35–39	8,848,819	–	180,244	7050.5	12,708	12,130	7943	4187
40–44	8,391,604	–	45,105	7050.5	3180	3035	1988	1048
45–49	7,678,041	–	3682	7050.5	260	248	162	86
Total	66,312,191	–	2,107,409	–	154,114	147,102	96,321	50,781

**Table 14:** Differentials in the net costs of influenza-related healthcare resulting from a 75% increase in vaccination coverage over the current coverage in the population aged 12 to 49 years with risk factors (million USD).

Costs	Diabetes	Uncontrolled Hypertension	Morbid Obesity	Chronic Renal Failure	Asthma	Pregnancy
Direct costs						
Diagnosis	−4.10	−2.95	−2.60	−0.36	−1.75	−2.97
Healthcare visits	−3.79	−2.87	−2.75	−0.32	−1.76	−2.98
Medications	−0.41	−0.32	−0.32	−0.04	−0.20	−0.35
Hospitalizations	−33.06	−23.06	−19.85	−2.66	−13.01	−22.08
Vaccination	5.52	4.45	5.17	0.54	3.53	5.39
Direct costs subtotal	−35.84	−24.75	−20.35	−2.84	−13.19	−22.99
Indirect costs						
Sick leave	−7.47	−5.44	−4.98	−0.60	−3.15	−5.35
Premature death	−1.14	−0.93	−1.85	−0.46	−0.09	−0.16
Indirect costs subtotal	−8.61	−6.37	−6.83	−1.06	−3.24	−5.51
Total costs	−44.45	−31.12	−27.18	−3.90	−16.43	−28.5

**Table 15:** Preventable cases of influenza, associated events, and net costs from the expansion of universal coverage to the population aged five–11 years and 50–59 years.

	5–11 Years	50–59 Years	Total
Preventions			
Influenza cases	671,461	199,500	870,961
Deaths	15	12	27
Events associated with preventable cases			
Healthcare visits	262,812	120,798	383,610
ER admission	154,143	33,024	187,167
Hospitalization	97,637	33,091	130,728
Net costs			
Direct costs			
Diagnosis	−13.27	−4.37	−17.64
Healthcare visits	−14.58	−4.48	−19.06
Medications	−2.59	−0.77	−3.36
Hospitalizations	−93.51	−41.66	−135.17
Vaccination	27.42	10.53	37.95
Direct costs subtotal	−96.53	−40.75	−137.28
Indirect costs			
Sick leave	−14.43	−8.07	−22.50
Premature death	−1.03	−0.99	−2.02
Indirect costs subtotal	−15.46	−9.06	−24.52
Total costs	−111.99	−49.81	−161.80

Net cost = costs − benefits. A negative cost value represents savings. ER = emergency room.

## Data Availability

All of the datasets generated and/or analyzed during the current study are available in the Bases de datos sobre defunciones (death databases) repository, http://www.dgis.salud.gob.mx/contenidos/basesdedatos/std_defunciones_gobmx.html (accessed on 20 February 2021), and the Egresos hospitalarios Secretaría de Salud (hospital discharges, Ministry of Health) repository, www.dgis.salud.gob.mx/contenidos/basesdedatos/da_egresoshosp_gobmx.html (accessed on 20 February 2021), with the exception of data from influenza cases obtained from SISVEFLU, which were analyzed with permission from Mexico’s General Directorate of Epidemiology (Ministry of Health) and are not publicly available in compliance with Mexico’s Personal Data Protection Law (http://www.diputados.gob.mx/LeyesBiblio/pdf/LFPDPPP.pdf (accessed on 20 February 2021)).

## Supplementary Materials

The following are available online at https://www.mdpi.com/2076-393X/9/3/286/s1, Figure S1: A call for a reform of the influenza immunization program in Mexico: Epidemiologic and economic evidence for decision making; Text S1: ICD-10 codes used for case selection.
