# Supplementary material for: A Call for a Reform of the Influenza Immunization Program in Mexico: Epidemiologic and Economic Evidence for Decision Making
Source: Vaccines (Basel). 2021 Mar 19;9(3):286. doi: 10.3390/vaccines9030286 (PMC8003748; doi:10.3390/vaccines9030286)
Supplement: Supplementary file 1 [file vaccines-09-00286-s001.pdf]

Roberto Tapia-Conyer[1,2], Miguel Betancourt-Cravioto[1], Jorge Abelardo Falcón-Lezama[1], Myrna María Alfaro-Cortes[1], Alejandra Montoya[1] and Rodrigo Saucedo-Martínez[1]

[1] Sociedad Mexicana de Salud Pública, Herschel 109, Anzures, Miguel Hidalgo, 11590, Ciudad de México, México
[2] Universidad Nacional Autónoma de México, Av. Universidad 3000, Circuito Escolar CU, Edificio B 1er Piso, Coyoacán, 04510, Ciudad de México, México
Corresponding author:Jorge Abelardo Falcón-Lezama Lago Zurich 245, Presa Falcón Piso 20, Col. Ampliación Granada, Miguel Hidalgo, 11529, Ciudad de México, México Phone: +52 (55) 5339 1796
Email: jfalcon@fundacioncarlosslim.org

Supplementary File

A call for a reform of the Influenza Immunization Program in Mexico: epidemiologic and economic evidence for decision making.

A call for a reform of the Influenza Immunization Program in Mexico: epidemiologic and economic evidence for decision making

Roberto Tapia-Conyer, Miguel Betancourt-Cravioto, Jorge Abelardo Falcón-Lezama*, Myrna María Alfaro-Cortes, Alejandra Montoya, Rodrigo Saucedo-Martínez

Background

Mexico's national vaccination schedule recommends yearly influenza immunization (free of charge at any public health facility) for several population groups during the influenza immunization season (October to April).

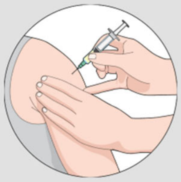

Coverage

9.9% (95% CI, 8.2 – 11.9)
to
33.9% (95% CI, 32.3 – 35.4)

*The range corresponds to the separate population groups noted below; these groups cannot be pooled together for analysis.

- **Children aged 6 to 59 months**
- **At-risk individuals aged 5 to 59 years** (obesity, hypertension, chronic renal failure, asthma, pregnant women, healthcare professionals)
- **Adults aged ≥60 years**

Objective

To provide guidance to the national influenza vaccination policy in Mexico by exploring the clinical effects and economic benefits of expanding the current national influenza vaccination program.

- **Children aged 6 to 59 months**
- **School-aged 5 to 11 years**
- **75% coverage in at-risk individuals aged 5 to 59 years**
- **Adults 50 to 59 years**
- **Adults aged ≥60 years**

Population

> 12 to 49 year olds with risk factors (diabetes mellitus, uncontrolled hypertension, chronic renal failure, morbid obesity (BMI ≥ 40 kg/m^2), pregnancy, and asthma)
> expanding the coverage to school-aged children (5 to 11 years old)
> expanding the coverage to 50 to 59 year olds with or without risk factors.

Analytical Methods

For the epidemiological analysis, the morbidity and mortality patterns were described for each season of the study period for both A and B-type circulating viruses. A decision-tree analytical model was used for economic analysis (eight scenarios based on SISVEFLU data).

Data sources

Data from the Influenza records (2009–2018) and Mexico's Influenza Surveillance System (SISVEFLU), death records (2010–2015) from the National Mortality Epidemiological and Statistical System, and discharge and hospitalization records (2010–2015) from the Automated Hospital Discharge System databases were used.

Results

Disease burden

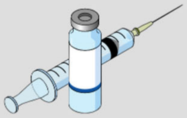

Using the surveillance criteria for influenza, SISVEFLU recorded a total of 390,862 suspected cases during the study period, of which 50,900 (13.03%) were confirmed with a diagnostic test.

Assuming 50% vaccine efficacy, expanding the vaccination program could prevent

- 7,142 to 671,461 influenza cases
- 7,615 to 262,812 health care visits
- 2,891 to 97,637 hospitalizations
- 1 to 15 deaths
- 2,886 to 154,143 ER admissions

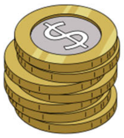

Net annual savings

11.7 to 111.9 million USD

(even after considering the investment needed for expanding the vaccination program)

Conclusions

Increasing the coverage to 75% in the population aged 12 to 49 years with risk factors, as well as expanding the universal vaccination coverage to adults 50 to 59 years old and school-aged children could potentially result in significant economic and health benefits. Efforts to improve the vaccination program aim to provide equal access to vaccination as a tool for disease prevention without limitations of sex, socio-economic status, and demographical conditions.

*Correspondence: jfalcon@fundacioncarlosslim.org
Vaccines, 2020, [volume], [pages]. DOI: [XX]

Supplement 1: General definition of influenza

Text S1. ICD-10 codes used for case selection

For influenza records from the databases of the National Mortality Epidemiological and Statistical System (SEED) [1] and the Automated Hospital Discharge System (SAEH) [2], the following codes from International Statistical Classification of Diseases and Related Health Problems, 10th revision, (ICD-10) [3] were used for the selection of cases: J09x, influenza due to certain identified influenza virus; J100, influenza with pneumonia, other influenza virus identified; J101, influenza with other respiratory manifestations, other influenza virus identified; J108, influenza with other manifestations, other influenza virus identified; J110, influenza with pneumonia, virus not identified; J111 influenza with other respiratory manifestations, virus not identified; and J18, influenza with other manifestations, virus not identified.

References for Supplement 1

1. Secretaría de Salud–SEED [Ministry of Health – SEED]. Available online: http://www.dgis.salud.gob.mx/contenidos/basesdedatos/std_defunciones_gobmx.html (accessed on 21 October 2020). Spanish.
2. Secretaría de Salud–SAEH [Ministry of Health – SAEH]. Available online: www.dgis.salud.gob.mx/contenidos/basesdedatos/da_egresoshosp_gobmx.html (accessed on 21 October 2020). Spanish.
3. World Health Organization. Available online: https://icd.who.int/browse10/2016/en (accessed on 21 October 2020).
